# An accurate algorithm to match imperfectly matched images for lung tumor detection without markers

**DOI:** 10.1120/jacmp.v16i3.5200

**Published:** 2015-05-08

**Authors:** Timothy Rozario, Sergey Bereg, Yulong Yan, Tsuicheng Chiu, Honghuan Liu, Vasant Kearney, Lan Jiang, Weihua Mao

**Affiliations:** ^1^ Department of Computer Science University of Texas at Dallas Richardson TX; ^2^ Department of Radiation Oncology University of Texas Southwestern Medical Center Dallas TX USA

**Keywords:** lung cancer, tumor tracking, markerless, template matching

## Abstract

In order to locate lung tumors on kV projection images without internal markers, digitally reconstructed radiographs (DRRs) are created and compared with projection images. However, lung tumors always move due to respiration and their locations change on projection images while they are static on DRRs. In addition, global image intensity discrepancies exist between DRRs and projections due to their different image orientations, scattering, and noises. This adversely affects comparison accuracy. A simple but efficient comparison algorithm is reported to match imperfectly matched projection images and DRRs. The kV projection images were matched with different DRRs in two steps. Preprocessing was performed in advance to generate two sets of DRRs. The tumors were removed from the planning 3D CT for a single phase of planning 4D CT images using planning contours of tumors. DRRs of background and DRRs of tumors were generated separately for every projection angle. The first step was to match projection images with DRRs of background signals. This method divided global images into a matrix of small tiles and similarities were evaluated by calculating normalized cross‐correlation (NCC) between corresponding tiles on projections and DRRs. The tile configuration (tile locations) was automatically optimized to keep the tumor within a single projection tile that had a bad matching with the corresponding DRR tile. A pixel‐based linear transformation was determined by linear interpolations of tile transformation results obtained during tile matching. The background DRRs were transformed to the projection image level and subtracted from it. The resulting subtracted image now contained only the tumor. The second step was to register DRRs of tumors to the subtracted image to locate the tumor. This method was successfully applied to kV fluoro images (about 1000 images) acquired on a Vero (BrainLAB) for dynamic tumor tracking on phantom studies. Radiation opaque markers were implanted and used as ground truth for tumor positions. Although other organs and bony structures introduced strong signals superimposed on tumors at some angles, this method accurately located tumors on every projection over 12 gantry angles. The maximum error was less than 2.2 mm, while the total average error was less than 0.9 mm. This algorithm was capable of detecting tumors without markers, despite strong background signals.

PACS numbers: 87.57.cj, 87.57.cp87.57.nj, 87.57.np, 87.57.Q‐, 87.59.bf, 87.63.lm

## I. INTRODUCTION

Lung cancer is the leading cause of cancer deaths in the United States. For a large percentage of lung cancer patients, radiotherapy is either the primary treatment modality or part of a combined treatment approach along with surgery and/or chemotherapy. The fundamental goal of radiotherapy is to deliver a tumoricidal radiation dose while simultaneously minimizing dose to critical structures and healthy normal tissue. Amplitude of tumor motion can range up to 2–3 cm depending on tumor locations and individual patients.^(1)^ Ways to negate the coverage of tumor motion have been investigated by Keall et al.^(1)^ and Jiang et al.^(2)^ Strategies such as beam gating and beam tracking^3^, ^4^ require precise tumor localization to be effective. Hence, a large planning margin may be required; however, large treatment volumes frequently limit the ability to deliver high doses due to deleterious side effects. An accurate method for identifying tumor motion on a daily basis for lung cancer treatment is paramount. The techniques to monitor tumor motions can be broadly classified into three categories: 1) implanted fiducial marker tracking or wireless transponders,^5^, ^6^, ^7^, ^8^ 2) tumor tracking via external or breathing surrogates,^2^, ^9^, ^10^, ^11^ and 3) markerless tumor tracking.^13^, ^14^, ^15^, ^16^, ^17^, ^18^

Using implanted fiducial markers to track tumors has been proven to be highly accurate,^5^, ^6^, ^7^, ^8^ but fiducial markers have two major drawbacks: 1) patient may be at risk from clinical complications like pneumothorax^19^, ^20^ and the possibility of infection from the implantation procedure; and 2) the possibility of the fiducial makers migrating to different locations in the lung.^2^, ^21^, ^22^ Using external surrogates to track lung tumor motion is a cost‐effective and safe technique because of its noninvasive procedure and absence of radiation doses, but the varying correlation between the tumor motion and the external surrogates during a single measurement session can lead to errors.^10^, ^11^, ^12^, ^23^ Tracking the motion of lung tumor using anatomic surrogates can be effective with well‐chosen surrogates such as the diaphragm.^(11)^ However, the relationship between the breathing surrogate and lung tumor can often vary inter‐and intrafractionally, leading to inaccurate results.^10^, ^11^, ^12^, ^23^

The problems caused due to implanted fiducial markers and internal and external surrogates can be overcome by using markerless lung tumor tracking techniques. Several techniques have been reported that track tumor motion by locating the tumor on each CBCT projection. Lewis et al.^(17)^ create a set of reference templates from 4D CT and compare them with rotational CBCT projections to determine the tumor motion. Creating a predefined set of templates can limit the field of view and restrict the location of tumor motion within a range defined by 4D CT. This technique employs kV imaging systems that provide better image quality and a wider view than electronic portal imaging devices.^24^, ^25^, ^26^ A markerless lung tumor detection technique has also been reported by Yang et al.,^(18)^ where a special set of digitally reconstructed radiographs (DRRs) are generated that removes the tumor and consists only of the thorax or upper abdomen anatomy. The CBCT projections are subtracted from the DRR, resulting in projections consisting predominantly of just the tumor. However, CBCT projections of thorax or upper abdomen contain respiratory‐induced image artifacts, such as blurring and scattering, that severely limit the ability to determine the tumor or affected regions. In addition, global discrepancies exist between DRRs and projections due to their different image orientations, scattering, and noises. Also, strong signals from the under‐ and overlying critical structures result in adversely affecting the comparison accuracy between DRRs and kV projection images.

The goal of this paper is to accurately locate the tumor on every kV projection using a markerless algorithm.

## II. MATERIALS AND METHODS

### A. Overall process

**Figure 1:**
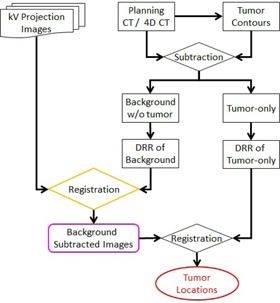
Flow chart of the complete tumor detecting procedure.

**Figure 2:**
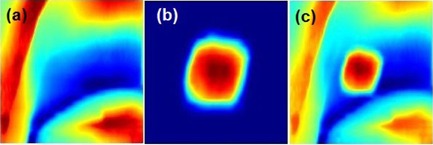
Sample DRRs: (a) background DRR, (b) tumor DRR, (c) composite DRR‐ tumor, and background DRR.

As shown in Fig. 1, preprocessing was performed in advance to generate two sets of DRRs to match with projection images in two steps. As part of the radiotherapy treatment planning process for lung cancer, a 4D CT procedure was performed. Maximum intensity projection images and average intensity projection images were generated from the 4D CT data and used in segmentation and image guidance, respectively. Once the contouring was completed, a binary text file was generated containing the CT voxel matrix (1 for tumor and 0 for background).^(18)^ Two subsets of anatomy structures, background without tumor and tumor only, were generated after the tumors were removed from the planning 3D CT or a single phase of planning 4D CT images using planning contours of tumors, as reported previously.^(18)^ Background DRRs and tumor DRRs were generated separately using a conventional ray‐tracing algorithm with trilinear interpolations (see Fig. 2 for sample DRRs). Two sets of DRRs were used in a two‐step process to locate the tumor on every frame. The first step of the process involved a rigid registration to match projection images with DRRs of background signals. The background DRRs and the projections were not perfectly matched, not only because the tumors were removed intentionally, but also due to global intensity discrepancies that existed due to their different image orientations, scattering, and noises. This adversely affected the comparison accuracy. A novel tile‐shifting technique was incorporated into the model to overcome imperfectly matched images. Details are described later in this section. The matched DRR was subtracted from the projection, resulting in the removal of under‐ and overlying anatomy, leaving the tumor isolated on the projection. A second rigid registration was performed between the subtracted projection and DRR containing only the tumor to identify the location of the tumor on the projection.

### B. Tiling technique

**Figure 3:**
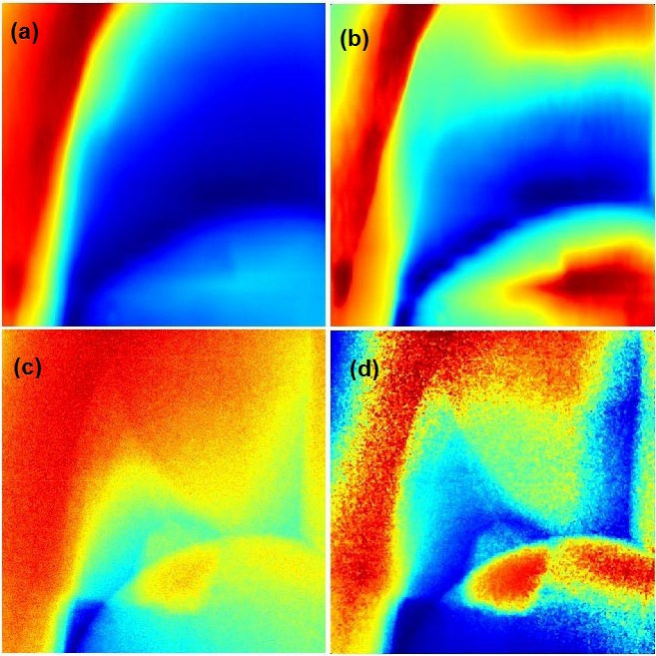
(a) and (c) are raw background DRR and projection image, respectively. (b) and (d) are CLAHE enhanced background DRR and projection image, respectively.

The tiling technique divided the global projection images and DRRs into a matrix of nonoverlapping small tiles before the initial matching was done. Similarities were evaluated by calculating normalized cross‐correlation (NCC) between corresponding tiles of projections and background DRRs (see Fig. 3). Equation (1) is referred to as NCC, where *P* represents a projection and *D* presents a DRR. Both P and D have tile matrix U×V (i.e., U columns and V rows tiles). For a tile, (u,v), its starting pixel is $\left(p_{u},p_{v}\right)$ on projection and is $\left(d_{u},d_{v}\right)$ on DRR, respectively. The NCC between projection tile (u,v) and DRR tile (u,v) will be

(1) $$s(u,v)=\frac{\sum_{x,y}\left[\{P(p_{u}+x,p_{v}+y)-{\bar{P}}_{u,v}][(D(d_{u}+x,d_{v}+y)-{\bar{D}}_{u,v}]\right\}}{\sqrt{\left\{\sum_{x,y}{\left[P(p_{u}+x,p_{v}+y)-{\bar{P}}_{u,v}\right]}^{2}\}\{\sum_{x,y}{\left[D(d_{u}+x,d_{v}+y)-{\bar{D}}_{u,v}\right]}^{2}\right\}}}.$$

${\bar{P}}_{u,v}$ and ${\bar{D}}_{u,v}$ are mean value of the tile (u,v) on projection and DRR, respectively.

The total NCC is the averaged NCC of the tiles as shown in Eq. (2):

(2) $$S=\frac{\sum_{u=1}^{U}\sum_{v=1}^{V}s(u,v)}{U-V}$$

**Figure 4:**
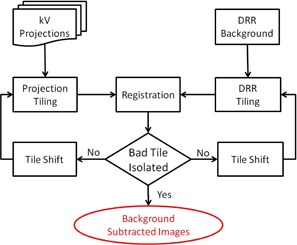
Flow chart of the tile‐shifting system to detect lung tumors on kV projections.

Tile configurations (i.e., global images) represented as a matrix of small tiles were defined by the user. Parameters such as size of projection images, tumor locations, and tumor sizes were considered when deciding the tile configuration. In our studies, we considered a tile configuration of 3 by 3 matrix of tiles. Image tiling allows NCC to be sensitive to local discrepancies that exist between corresponding DRR and projection tiles, and image artifacts that are caused by respiration induced tumor motion. Care was taken when selecting the size of the tiles such that the tumor fit within a single tile. This allows for better contrast of the tumor when contained within the tile, while reducing the search for the location of the tumor to approximately a single tile. The tile sizes are approximately between 1.2 to 1.5 times the tumor sizes. The example considered in this paper assumes the tumor to be approximately 140 by 140 pixels and the tile size approximately 168 by 168 pixels. However, prior to the image tile‐shifting process we performed image enhancement processing. Specifically, a contrast enhancement method was applied to the DRR and projection since the background DRR values were not linearly correlated to the projection image intensities. Therefore, contrast limited adaptive histogram equalization (CLAHE)^(27)^ was applied to correct the gray levels of the background DRR and projection to improve their efficacy (see Fig. 4 for illustration). CLAHE divided the background DRR and projection into a matrix of contextual regions and performed local contrast manipulation using histogram equalization on each of these regions. The contrast enhancement of the image was the cumulative optimized contrasts of the individual regions. This process of partitioning the image into regions may have introduced background noise. CLAHE overcomes this drawback by adding a contrast‐limiting factor that limits the number of pixels in each bin and by equally redistributing the extra pixels over the histogram (see Fig. 4).

### C. Tile shifting

After the global images (projections and DRRs) are divided into tiles, the tile configuration might be modified by globally shifting the starting point of one image. For example, $\left\{(p_{u},p_{v})\right\}$ might be shifted together by adding certain number of pixels to each direction. The starting point of DRR tiles might be shifted differently. As a result, the similarity would be compared on different regions of projection and DRR. In this way, the registration evaluated different trials. Figure 4 shows the flow chart of the tile‐shifting technique.

**Figure 5:**
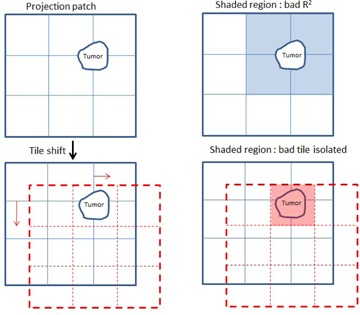
Shifting tiles to move the tumor within a single tile.

Since background DRRs and projections were not perfectly matched due to the absence of tumors in background DRRs, it is known that there would be a region with low similarity. The purpose of this method was to shift the tile configuration and move the tumor region into one single tile, as illustrated in Fig. 5. As long as the tumor was isolated to a single tile, this tile would have low similarity measure, while the remaining tiles had high similarity. The tile with the low similarity measure (i.e., the tile containing the tumor) was defined as bad tile. The bad tile was ignored when calculating the total NCC to improve matching between the globally shifted projection and background DRR.

**Figure 6:**
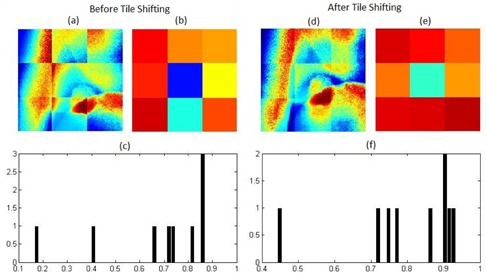
(a) Tiled kV projection before tile‐shifting; (b) R^2^ image of tiles before shifting; (c) R^2^ plot on initial kV projection and corresponding DRR tiles. Similarly, (d),(e), and (f) show the tiled shifted kV projection, R^2^ image, and plot of tiles on kV projection and corresponding DRR tiles. The image tiles are not continuous because every tile has been normalized individually also for the purpose of presenting tiling configuration.

Figure 6 shows projection images and corresponding NCC results for every tile. The tumor appears to be present in more than one tile (Fig. 6(a)). This is also seen in the tile‐wise cross‐correlation coefficient (R^2^) image (Fig. 6(b)) and histogram plot (Fig. 6(c)) where there was more than one bad tile. After shifting the tiles, the tumor appeared to be within a single tile (see Fig. 6(d)). The similarity measure for that tile was low, as indicated by the blue colored tile in Fig. 6(e), while the rest of the tiles had high similarity measures as, seen in Fig. 6(f). Hence the tumor was isolated to a single tile.

### D. Subtracting background

A pixel‐based linear transformation was determined using bilinear interpolations of tile matching results on projections that transformed pixel intensities such that the tile‐induced boundaries were removed. The transformation between the bad‐tile pair was determined by bilinearly interpolating the transformations from the centers of nearby tiles. Once the DRR was transformed to match the projection, it was then subtracted from the projection image. This resulted in the tumor emerging as the major difference on the projection.

### E. Locate tumors

Finally, the lung tumor was located on the projection image using the tumor DRR as template after the underlying and overlying anatomy had been subtracted. A second rigid registration was performed by calculating the NCC between the subtracted global projection and the tumor DRR. Thus the tumor locations were determined in two dimensions.

### F. Evaluations

The overall accuracy of this method was evaluated using phantom studies. A sophisticated respiratory torso phantom was built with a phantom (RS‐1500) (Radiology Support Devices, Long Beach, CA) consisting of a complex humanoid torso including lungs, ribcage/chest‐wall bone, skin, and subdermis. A tumor was simulated by a cylindrical bulk of pliable bolus with dimensions of approximately 3 cm in diameter by 3 cm in height. The heart was simulated by a sphere filled with water. The tumor was driven by a programmable 4D motion platform (Washington University, St Louis, MO), which could apply arbitrary motion patterns. Radiation opaque markers were implanted and used as ground truth for tumor positions. Dual orthogonal kV fluoro images were acquired on a Vero (BrainLAB, AG, Feldkirchen, Germany) for dynamic tumor tracking mode. The imager had a pixel size of 0.4 mm with a source‐to‐detector distance of 1876 mm, while the source‐to‐axis distance was 1000 mm.

## III. RESULTS & DISCUSSION

Twenty‐four sets of kV fluoro images were acquired over 12 gantry angles and about 1000 raw projections were analyzed (approximately 83 images per gantry angle). Three sphere‐shaped internal fiducial markers with a diameter of 5.5 mm were implanted next to the simulated tumor. Marker positions were easily detected using a pattern (template) matching method.^(28)^ The marker positions were used as ground truth for tumor positions. In order to minimize the effects of markers, a simple and efficient method was used to remove markers from original projection images and CT images. The markers were first located using the aforementioned pattern matching method;^(28)^ next we placed a 0/1 grid (mask) over the marker locations and modified pixel intensities by averaging surrounding pixels values. Each step modifies pixel intensities of the boundary of the marker; we continue this process while reducing the radius of the makers at each step till the markers vanish. Basically, the holes were filled gradually by interpolation of intensities of surrounding existing pixels, so that the holes were filled smoothly. All of the studies were performed on the data after markers were removed.

**Figure 7:**
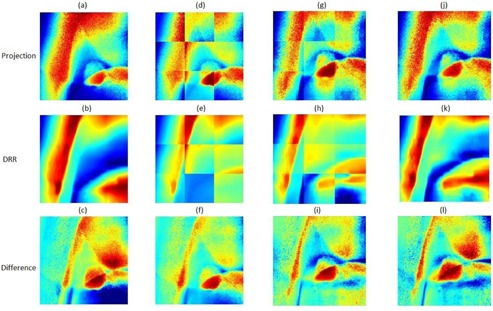
Nontiled kV projection (a) matched with DRR (b) and their differences (c). Tiled kV projection (d) matched with DRR (e) and their differences (c). Similarly, columns 3 and 4 follow columns 2 and 1, respectively, after shifting matched kV projection and DRR.

The effectiveness of our tiling method is shown in Fig. 7 while comparing with the traditional method for finding lung tumors in projections with strong anatomical signals. The background DRRs (Figs. 7(e) and 7(h)) are transformed more accurately to the projection image level than in Figs. 7(b) and 7(k), which do not tile the images into matrices of small tiles. As a result, the background noise was removed and the tumor emerged in the subtracted projection more effectively than in the traditional method (see Figs. 7(f) and 7(i), respectively).

**Figure 8:**
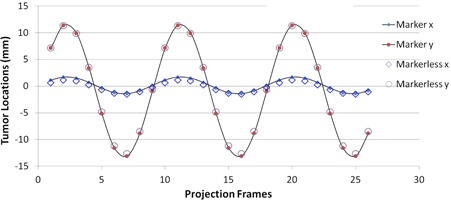
Comparison of tumor tracking results between markerless method and markers detection technique.

Figure 8 illustrates the results of lung tumor motion on kV fluoro images at an angle of 105°. Results of our markerless method were compared with those of the implanted tumor markers. Table 1 lists the maximum and average difference in tumor locations between our markerless method and the implanted tumor markers for the 24 sets of kV fluoro images. The maximum difference is less than 2.2 mm, while the total average difference is less than 0.8 mm. Although simulated heart and bony structures introduced strong signals superimposed on tumors at some angles, our method accurately located tumors on every projection over 12 gantry angles. It also should be noted that this markerless method was based on CT scans with a slice thickness of 3 mm.

**Table 1:** Summary of markerless tumor tracking results compared with marker detection technique.

*Gantry Angle (°)*	*kV Imaging Angle (°)*	*Max Difference (mm)*	*Average Difference (mm)*
0	45	0.7	0.5
0	315	1.4	1.1
30	75	2.2	1.5
30	345	0.6	0.4
60	15	0.5	0.3
60	105	1.1	0.7
90	45	1.2	1.0
90	135	0.9	0.7
120	75	2.2	1.6
120	165	0.7	0.5
150	105	2.0	1.3
150	195	0.9	0.7
180	135	0.9	0.7
180	225	0.9	0.7
210	165	0.8	0.5
210	255	1.5	0.9
240	195	0.7	0.4
240	285	1.9	1.4
270	225	1.3	1.2
270	315	1.6	1.1
300	255	1.0	0.8
300	345	0.8	0.5
330	15	0.5	0.4
330	285	2.1	1.6

## IV. CONCLUSIONS

This algorithm is capable of accurately detecting lung tumors without implanted markers despite the presence of strong underlying and overlying anatomy, and the global discrepancies that exist between DRRs and projections on phantom studies. The next step of this project is to acquire kV projections of patient data and study how this method performs on it. Ideally, the tumor detection method described in this paper would yield the best results when applied to DRRs and projections of perfectly aligned patients. Although the DRRs are generated using the same isocenter that is used in the generation of projections, there will be some discrepancies. Small local differences may be neglected due to the nature of NCC similarity measurement. However, nonrigid deformation of the anatomy will be necessary to address large differences.

## ACKNOWLEDGMENTS

This project has been partially supported by an Elekta Research Grant.
